# Young para-athletes display more hedonic well-being than people with disabilities not taking part in competitive sports: insights from a multi-country survey

**DOI:** 10.3389/fpsyg.2023.1176595

**Published:** 2023-07-13

**Authors:** Luca Puce, Carlo Biz, Mariachiara Cerchiaro, Davide Scapinello, Luca Giarrizzo, Carlo Trompetto, Lucio Marinelli, Khaled Trabelsi, Mohammad Hossein Samanipour, Nicola Luigi Bragazzi, Pietro Ruggieri

**Affiliations:** ^1^Department of Neuroscience, Rehabilitation, Ophthalmology, Genetics, Maternal and Child Health (DINOGMI), University of Genoa, Genoa, Italy; ^2^Orthopedics and Orthopedic Oncology, Department of Surgery, Oncology and Gastroenterology (DiSCOG), University of Padova, Padova, Italy; ^3^Istituto di Ricovero e Cura a Carattere Scientifico (IRCCS) Ospedale Policlinico San Martino, Genova, Italy; ^4^Institut Supérieur Du Sport et de L'Éducation Physique de Sfax, Université de Sfax, Sfax, Tunisia; ^5^Research Laboratory: Education, Motricité, Sport et Santé, EM2S, LR19JS01, Sfax University, Sfax, Tunisia; ^6^Department of Sport Science, Imam Khomeini International University, Qazvin, Iran; ^7^Laboratory for Industrial and Applied Mathematics (LIAM), Department of Mathematics and Statistics, York University, Toronto, ON, Canada

**Keywords:** global well-being, hedonic well-being, sports-related well-being, disabled athletes, para-athletes, multi-country survey

## Abstract

Hedonic well-being relates to how individuals experience and rate their lives. People with disabilities due to their pathology may more frequently suffer from anxiety and depressive disorders than their able-bodied counterparts. Sports participation is an essential way to cope with disability. On the other hand, compared with their able-bodied peers, para-athletes undergo a unique series of stressors. Little is known in terms of hedonic well-being in this specific population. We present the results of a multi-country survey of self-perceived hedonic well-being by para-athletes of different sports disciplines and a control group (disabled individuals not playing competitive sports), using the “Psychological General Well-Being Index” (PGWBI). We included 1,208 participants, aged 17.39 years, 58.4% male, 41.6% female, and 70.3% para-athletes. Para-athletes exhibited higher well-being than disabled people, for all domains of the PGWBI scale. The nature of disability/impairment was significant, with those with acquired disability reporting lower well-being. Those taking part in wheelchair basketball, para-athletics, and para-swimming competitions had a higher likelihood of reporting well-being, whereas those engaged in wheelchair rugby exhibited lower well-being compared with controls. This large-scale investigation can enable a better understanding of the self-perceived hedonic well-being of disabled people.

## 1. Introduction

Human well-being, as a complex, multi-dimensional construct, consists of hedonic and eudaimonic dimensions (Ryan and Deci, 2001). The former, also known as subjective well-being (SWB), relates to how individuals live and evaluate aspects of their lives (Diener, 1984), in terms of overall and domain-specific life satisfaction, and positive and negative affect, the balance of which is known as happiness. Eudaimonic well-being, also known as psychological and social well-being, is, instead, related to self-acceptance, meaningful connections, autonomy, environmental mastery, purpose in life, and personal growth (Kim et al., 2022). Even if these two models of well-being (hedonic/SWB and eudaimonic/psychological and social well-being) are theoretically diverging, they partially overlap (Ryan and Deci, 2001). Recently, these concepts have been adapted to the elite sports world (Lundqvist, 2011): well-being, as experienced by athletes is complex and nuanced (Lundqvist and Sandin, 2014). The sports arena can be, indeed, either challenging or rewarding, providing new opportunities and situations to explore (Filbay et al., 2019; Giles et al., 2020). On the one hand, athletes can connect with peers and the public, having stimulating interactions that improve self-confidence and control of their environment (Jones et al., 1994). On the other hand, besides success, they can experience failure, and they have to undergo heavy training programs, and nutritional restrictions daily with the risk of incurring overtraining, injury, and burnout (Rice et al., 2016). Also, they have to cope with a variety of challenges, and stressors (Mack et al., 2012) that can put their mental health and well-being at risk (McLoughlin et al., 2021). People with disabilities usually report poorer well-being due to their underlying conditions (Tilly, 2008; Krahn et al., 2015; Tough et al., 2017), even if they can overcome adverse situations by adopting coping strategies (Bahmani et al., 2022). Practicing sports and exercising can help face disability-related stressors (Puce et al., 2017; Ascione, 2018; Kiuppis, 2018; Puce et al., 2019), even though, compared with their able-bodied counterparts, athletes with disabilities and para-athletes encounter a unique series of stressors, from discrimination, retaliation, and stigmatizing situations, including demeaning attitudes and unprofessional coaching, to physical access, communication, or economic-financial barriers (Iezzoni, 2009; Jefferies et al., 2012). If sport and physical activity are well-known to facilitate inclusion, favor social acceptance, and improve self-esteem (Trigueros et al., 2021), there is a paucity of data concerning well-being from a hedonic perspective in this specific population (Macdougall et al., 2015). The existing scholarly literature reports contrasting results and rarely compares para-athletes with subjects with disabilities non-practicing competitive para-sports, providing little evidence on the psychological benefits of competitive sports for individuals with disabilities. Furthermore, research is limited to specific types of disability/impairment, as well as to selected para-sports disciplines, settings, and geographic contexts, with small sample sizes. Therefore, given this dearth of information, this study was conducted to fill in this gap of knowledge. Our aim was to test the specific hypothesis that the practice of competitive sports would have a positive impact on the well-being of the participants. In this cross-sectional study, we present the results of a multi-country survey of self-perceived hedonic well-being by para-athletes of different sports disciplines and a control group (people with disabilities, not playing competitive sports). Our main independent variable was competitive sporting practice, but we also looked at the participant’s sex/gender, age, type of impairment, way of administration of the survey, and country of provenience. We believe that a large-scale investigation can enable a better understanding of the self-perceived hedonic well-being and related needs of people with disabilities and whether competitive sports-associated stressors can affect the quality of life-related outcomes.

## 2. Materials and methods

### 2.1. Study participants

1,408 participants were enrolled in the present study. Due to missing data (95 among para-athletes and 105 among disabled people), 200 questionnaires were discarded. Data about 1,208 participants (85.8%) were retained. Socio-demographic features of the discarded and retained groups did not differ in a statistically significant way.

Included subjects were aged 17.39 ± 3.44 (median 17) years, 705 (58.4%) male and 503 (41.6%) female, were considered in the present analysis. 849 (70.3%) were para-athletes, and the remainder 359 (29.7%) were disabled people not taking part in competitive sports. According to para-sport discipline, 105 (12.4%) were engaged in para-athletics, 78 (9.2%) in wheelchair rugby, 66 (7.8%) in boccia, 61 (7.2%) in blind football, 27 (3.2%) in goalball, 31 (3.7%) in judo, 353 (41.6%) in para-swimming, 33 (3.9%) in para-table-tennis, 20 (2.4%) in para-sailing, and 75 (8.8%) in wheelchair basket. In terms of geographic provenience, 294 (24.3%) were from Western Europe, 449 (37.2%) from Southern Europe, 381 (31.5%) from Northern Europe, and 84 (7.0%) from Central-Eastern Europe. 819 (67.8%) and 389 (32.2%) were from urban and rural environments, respectively. Disability/impairment was congenital in 830 (68.7%) cases and acquired in 378 (31.3%) cases. It was musculoskeletal in 370 (30.6%) cases, sensory in 148 (12.3%) cases, neurological in 589 (48.8%) cases, and intellectual/relational in 101 (8.4%) cases. In 362 cases (30.0%), the participant required help in filling in the questionnaire. 274 (22.7%) filled in the questionnaire during the competitive period.

### 2.2. Procedure

This study was designed as a case–control, questionnaire-based, cross-sectional survey. Its major findings have been reported in accordance with the “Strengthening the Reporting of Observational Studies in Epidemiology” (STROBE) checklists (Vandenbroucke et al., 2007). A portion of the cases were young para-athletes who participated in the “European Para Youth Games” (EPYG), which took place from 9 to 15 October 2017, in Liguria, Italy. EPYG is an international, competitive, and multisport competition for youth athletes with disabilities (aged 12–23 years) organized and regulated by the International Paralympic Committee (IPC). This event, which takes place every 2 or 3 years, is hosted each time in a different European country. One month before the event, the notification and information on the questionnaires were published on the official web page of the event and sent by e-mail to the federations of the various para-sports disciplines. This email and the web page of the event contained a link to a web form developed using the Google Forms cloud-based service, consisting of an explanatory note, a form in which subjects were asked to express their willingness or unwillingness to participate in the survey, the questionnaires to be filled in, and an additional questionnaire containing help information. The same questionnaires and explanatory notes were administered to a reference sample of disabled young people who do not practice competitive sports, of similar age, by sending the link to the event web page via email to rehabilitation clinics based in the European countries participating in the event. To reach the largest possible number of both para-athletes and disabled individuals non-practicing sports, the web form remained open until July 2019 for both populations. The study protocol was approved by the local ethics committee and published in a peer-reviewed international scholarly journal (Puce et al., 2017). The study was prospectively registered within the ISRCTN (“International trial registry”) repository (registration code ISRCTN14389453).

### 2.3. Psychometric tool

Among the various tools for calculating hedonic well-being (Cooke et al., 2016), the “Psychological General Well-Being Index” (PGWBI) was chosen (Dupuy, 1984). This represents a commonly employed and reliable survey index that measures self-perceived psychological health and well-being over the past 4 weeks (Paleari et al., 2021). This tool was developed in the United States and has subsequently been tested in other countries on large samples of the general population, as well as specific patient and population groups, including athletes and sportspeople with disabilities (Puce et al., 2019; Maugeri et al., 2020). The questionnaire score results in six domains, and each domain consists of three to five items: i.e., 3 items for depression, self-control, and general health, 4 items for vitality and positive well-being, and 5 items for anxiety.

For example: “Have you been bothered by nervousness or your “nerves” during the past month?” is a specific anxiety rating question and requires an answer ranging from “Extremely so – to the point where I could not work or take care of things” for severe anxiety to “Not at all” for no anxiety. Furthermore, each item is rated on a Likert-like scale from 0 (the most negative option) to 5 (the most positive option); the global summary score reflects severe distress between 0 and 60 while reflects levels of moderate distress and positive well-being between 61 and 72 and 73 and 110 points, respectively, with a maximum of 110 points representing the best attainable level of hedonic well-being.

### 2.4. Statistical analysis

An *a priori* sample size and power analysis was conducted, using G*Power software (version 3.1.9.7, Heinrich Heine University Düsseldorf, Düsseldorf, Germany). In the existing scholarly literature, only a few studies have compared the quality of life in people with disabilities practicing or not practicing sports, reporting large effect sizes (Fiorilli et al., 2013; Dantas et al., 2022). To detect a large effect size, with an alpha error probability of 0.05 and a power of 0.80, a sample of 102 participants would have been enough, whereas to detect a small effect size, with the same parameters, a sample of 620 individuals would have been needed.

Once collected, questionnaire data were analyzed in terms of skewness, kurtosis, and normal distribution. Descriptive statistics were carried out, by computing means, standard deviations, and medians. The psychometric properties of the tool, including the Cronbach’s alpha, were also assessed: overall, the Cronbach’s alpha coefficient was 0.93, with the coefficient varying from 0.63 to 0.80 for each domain (namely, the anxiety scale has a coefficient of 0.74, the depressed mood scale a coefficient of 0.80, the positive well-being scale a coefficient of 0.70, the self-control scale a coefficient of 0.76, the general health scale a coefficient of 0.65, and the vitality scale a coefficient of 0.63). These values are well in line with those reported in the literature, confirming the reliability of the data collected.

Univariate and multivariate analyses were conducted to shed light on the determinants of well-being among athletes with disabilities. Different models were run, and the best one was selected based on the deviance, Akaike Information Criterion (AIC), Bayesian Information Criterion (BIC), McFadden’s *R*^2^, Nagelkerke’s pseudo-*R*^2^, Cox and Snell’s *R*^2^, and overall model chi-squared test. Effect sizes were also computed. All analyses were done using the “Statistical Package for Social Sciences” (SPSS for Windows, version 26.0, Armonk, IBM, NY, United States). We test at an alpha level of 0.05.

## 3. Results

### 3.1. Hedonic well-being

The scores of the PGWBI broken down according to each domain for the entire study population are shown in Table 1. 461 (38.2%) of the population exhibited well-being, whilst 206 (17.1%) and 541 (44.8%) displayed moderate and severe distress, respectively. Stratifying according to the type of population, 419 (49.4%) para-athletes reported well-being, 161 (19.0%), and 269 (31.7%) exhibited moderate and severe distress. Forty-two (11.7%) people with disabilities (not participating in competitive sports) reported well-being, 45 (12.5%) had moderate distress, and 272 (75.8%) exhibited severe distress. Differences for all variables under study (overall PGWBI and all domain scores) between the two populations are significant (*p* < 0.001), with large effect sizes (Table 2).

**Table 1:** The “Psychological General Well-Being Index” (PGWBI) scores are presented, broken down according to PGWBI domains in the overall study population.

PGWBI domain	Overall study population (*n* = 1,208)
Anxiety score	15.41 ± 4.82
Depressed mood score	9.38 ± 3.63
Positive well-being score	10.56 ± 3.99
Self-control score	8.59 ± 3.47
Vitality score	12.25 ± 3.73
General health score	9.91 ± 3.28
PGWBI summary score	66.10 ± 18.35

**Table 2:** The “Psychological General Well-Being Index” (PGWBI) scores for each PGWBI domain, broken down according to the type of population (disabled people, *n* = 359, versus para-athletes, *n* = 849).

PGWBI domain	People with disabilities (*n* = 359)	Para-athletes (*n* = 849)	Effect size
Anxiety score	12.14 ± 4.28	16.79 ± 4.34	1.08 [95%CI 0.95–1.21]
Depressed mood score	7.51 ± 2.96	10.18 ± 3.60	0.78 [95%CI 0.65–0.91]
Positive well-being score	8.07 ± 2.94	11.62 ± 3.90	0.98 [95%CI 0.85–1.10]
Self-control score	6.72 ± 2.70	9.39 ± 3.46	0.82 [95%CI 0.69–0.95]
Vitality score	9.75 ± 2.90	13.30 ± 3.54	1.06 [95%CI 0.93–1.19]
General health score	7.92 ± 3.00	10.75 ± 3.02	0.94 [95%CI 0.81–1.07]
PGWBI summary score	52.11 ± 15.16	72.02 ± 16.25	1.25 [95%CI 1.12–1.38]

### 3.2. Determinants of hedonic well-being

In the univariate analyses (Tables 3, 4), concerning the PGWB overall score, para-athletics and para-swimming reported the highest value, while wheelchair basketball yielded an intermediate value and the remainder the lowest one. Besides para-sports disciplines, also the nature of the disability/impairment (congenital versus acquired) was found to be statistically significant. In the multivariate analysis, the PGWB score was dichotomized into two categories: being positive and experiencing moderate-to-severe psychological distress. The nature of disability/impairment was statistically significant, with those with acquired disability reporting lower well-being (OR 0.19 [0.13–0.26], *p* < 0.001). Those taking part in wheelchair basketball, para-athletics, and para-swimming competitions had a higher likelihood of reporting well-being (OR 51.51 [95%CI 26.51–104.0.3], *p* < 0.001; OR 47.93 [95%CI 26.66–89.55], *p* < 0.001; and OR 46.51 [95%CI 30.37–72.40], *p* < 0.001), whereas those engaged in wheelchair rugby exhibited lower well-being when compared to their counterparts not participating in competitive sports (OR 0.46 [95%CI 0.22–0.91], *p* = 0.032) (Table 5).

**Table 3:** The “Psychological General Well-Being Index” (PGWBI) scores for each PGWBI domain, broken down according to socio-demographic/clinical variables.

Socio-demographic/clinical variable	Anxiety score	Depressed mood score	Positive well-being score	Self-control score	Vitality score	General health score	PGWBI summary score
Sex/gender							
Female (*n* = 503)	15.41 ± 4.67	9.53 ± 3.59	10.49 ± 3.97	8.72 ± 3.37	11.89 ± 3.63	9.86 ± 3.38	65.89 ± 18.45
Male (*n* = 705)	15.41 ± 4.92	9.28 ± 3.67	10.61 ± 4.01	8.51 ± 3.54	12.50 ± 3.79	9.94 ± 3.20	66.26 ± 18.29
Age (years)							
11–17 (*n* = 638)	16.07 ± 4.87	9.75 ± 3.67	11.02 ± 3.98	8.93 ± 3.51	12.61 ± 3.73	10.16 ± 3.30	68.54 ± 18.49
18–23 (*n* = 570)	14.67 ± 4.65	8.97 ± 3.55	10.05 ± 3.94	8.22 ± 3.39	11.84 ± 3.70	9.62 ± 3.24	63.37 ± 17.81
Disability type							
Muskuloskeletal (*n* = 370)	15.52 ± 4.87	9.42 ± 3.59	10.44 ± 4.19	8.60 ± 3.59	12.49 ± 3.80	10.37 ± 3.32	66.84 ± 18.79
Sensory (*n* = 148)	14.95 ± 4.61	8.64 ± 3.80	10.39 ± 3.87	8.51 ± 3.09	12.03 ± 3.55	9.51 ± 3.12	64.01 ± 17.17
Neurological (*n* = 589)	15.24 ± 4.78	9.21 ± 3.55	10.30 ± 3.81	8.46 ± 3.42	11.88 ± 3.65	9.49 ± 3.20	64.59 ± 17.77
Intellectual-relational (*n* = 101)	16.62 ± 4.99	11.36 ± 3.38	12.81 ± 3.77	9.51 ± 3.76	13.82 ± 3.80	11.22 ± 3.27	75.34 ± 19.01
Congenital disability (*n* = 830)	16.16 ± 4.58	9.87 ± 3.56	11.20 ± 3.93	9.06 ± 3.48	12.92 ± 3.59	10.28 ± 3.21	69.48 ± 17.63
Acquired disability (*n* = 378)	13.77 ± 4.94	8.33 ± 3.58	9.17 ± 3.76	7.58 ± 3.23	10.77 ± 3.63	9.09 ± 3.29	58.70 ± 17.71

**Table 4:** The “Psychological General Well-Being Index” (PGWBI) scores for each PGWBI domain, broken down according to para-sports discipline.

Para-sports discipline	Anxiety score	Depressed mood score	Positive well-being score	Self-control score	Vital score	General health score	PGWBI summary score
Para-athletics (*n* = 105)	18.54 ± 4.16	7.51 ± 2.96	13.42 ± 3.44	11.16 ± 2.62	14.42 ± 3.31	12.13 ± 2.54	82.03 ± 14.06
Wheelchair rugby (*n* = 78)	14.27 ± 5.22	12.35 ± 2.17	7.12 ± 3.28	4.77 ± 2.01	12.68 ± 4.37	10.44 ± 3.02	54.59 ± 6.26
Boccia (*n* = 66)	12.88 ± 2.97	5.32 ± 1.98	10.53 ± 2.33	7.85 ± 1.96	10.94 ± 2.16	8.09 ± 1.92	58.48 ± 8.69
Blind football (*n* = 61)	16.36 ± 4.19	8.20 ± 2.01	8.66 ± 3.05	5.80 ± 2.05	12.20 ± 3.46	7.89 ± 2.59	56.98 ± 6.83
Goalbal (*n* = 27)	13.78 ± 2.29	6.08 ± 1.70	9.89 ± 3.43	7.89 ± 2.49	12.37 ± 2.76	8.19 ± 1.90	59.07 ± 4.59
Judo (*n* = 31)	13.71 ± 3.38	6.96 ± 2.92	10.94 ± 3.24	7.45 ± 2.28	11.84 ± 3.12	7.87 ± 2.11	58.58 ± 6.07
Para-swimming (*n* = 353)	18.33 ± 3.69	6.77 ± 2.67	13.25 ± 3.39	11.20 ± 2.70	14.04 ± 3.47	11.99 ± 2.23	81.19 ± 13.32
Para-table-tennis (*n* = 33)	14.52 ± 4.06	12.37 ± 2.13	9.27 ± 3.07	6.33 ± 2.10	13.18 ± 3.13	7.24 ± 2.56	56.64 ± 6.04
Para-sailing (*n* = 20)	14.85 ± 3.23	7.20 ± 4.21	7.90 ± 2.15	6.70 ± 3.45	11.70 ± 3.53	8.40 ± 4.04	56.75 ± 7.08
Wheelchair basketball (*n* = 75)	17.39 ± 3.63	12.11 ± 2.11	12.39 ± 3.33	10.81 ± 2.61	13.32 ± 3.25	12.21 ± 2.29	78.21 ± 12.31

**Table 5:** Multivariable regression model shedding light on the predictors of the “Psychological General Well-being Index” (PGWBI) summary score.

		95% confidence interval						95% confidence interval	
Predictor	Estimate	Lower	Upper	SE	*Z*-value	value of *p*	Odds ratio	Lower	Upper
Age	−0.0295	−0.0664	0.00804	0.0188	−1.5662	0.117	0.971	0.936	1.008
Sex/gender									
Male (vs. female)	0.1067	−0.1820	0.39666	0.1475	0.7237	0.469	1.113	0.834	1.487
Country/geographic provenience									
Southern Europe (vs. Western Europe)	0.3131	−0.0525	0.68159	0.1871	1.6732	0.094	1.368	0.949	1.977
Northern Europe (vs. Western Europe)	0.2823	−0.0881	0.65415	0.1892	1.4919	0.136	1.326	0.916	1.924
Central-Eastern Europe (vs. Western Europe)	0.0690	−0.5292	0.65391	0.3012	0.2291	0.819	1.071	0.589	1.923
Socio-economic status									
Urban (vs. rural)	−0.1149	−0.4153	0.18483	0.1530	−0.7511	0.453	0.891	0.660	1.203
Type of disability									
Sensory (vs. musculoskeletal)	−0.0981	−0.7175	0.52193	0.3158	−0.3108	0.756	0.907	0.488	1.685
Neurological (vs. musculoskeletal)	0.0171	−0.3197	0.35511	0.1720	0.0992	0.921	1.017	0.726	1.426
Intellectual-relational (vs. musculoskeletal)	0.3582	−0.3005	1.02543	0.3380	1.0597	0.289	1.431	0.740	2.788
Administration									
With aid (vs. without aid)	0.3106	−0.0899	0.71111	0.2042	1.5214	0.128	1.364	0.914	2.036
Classification of disability									
Acquired (vs. congenital)	−1.6894	−2.0280	−1.36223	0.1697	−9.9557	< 0.001	0.185	0.132	0.256
Timing of administration									
During the competition (vs. outside of the competition)	0.2147	−0.1785	0.61103	0.2012	1.0674	0.286	1.240	0.836	1.842
Para-sports discipline									
Para-athletics (vs. no competitive para-sport)	3.8698	3.2831	4.49479	0.3083	12.5514	< 0.001	47.931	26.658	89.549
Wheelchair rugby (vs. no competitive para-sport)	−0.7722	−1.5176	−0.09251	0.3611	−2.1384	0.032	0.462	0.219	0.912
Boccia (vs. no competitive para-sport)	0.3981	−0.2405	1.02024	0.3209	1.2407	0.215	1.489	0.786	2.774
Blind football (vs. no competitive para-sport)	−0.2145	−0.8851	0.41560	0.3301	−0.6496	0.516	0.807	0.413	1.515
Goalball (vs. no competitive para-sport)	−0.4001	−1.4428	0.57157	0.5099	−0.7846	0.433	0.670	0.236	1.771
Judo (vs. no competitive para-sport)	0.5570	−0.3160	1.41534	0.4403	1.2652	0.206	1.745	0.729	4.118
Para-swimming (vs. no competitive para-sport)	3.8396	3.4135	4.28223	0.2215	17.3371	< 0.001	46.505	30.372	72.401
Para-table-tennis (vs. no competitive para-sport)	−0.4244	−1.3970	0.43113	0.4603	−0.9220	0.357	0.654	0.247	1.539
Para-sailing (vs. no competitive para-sport)	−0.4592	−1.7716	0.64123	0.6013	−0.7637	0.445	0.632	0.170	1.899
Wheelchair basketball (vs. no competitive para-sport)	3.9418	3.2773	4.64466	0.3480	11.3275	< 0.001	51.510	26.505	104.028

SE, standard error.

## 4. Discussion

In the present study, we found that para-athletes reported higher hedonic SWB levels than their counterparts with disabilities not practicing competitive para-sports. In the univariate analyses, concerning the PGWBI overall score, para-athletics and para-swimming reported the highest value, while wheelchair basketball yielded an intermediate value and the remainder the lowest one. Besides para-sports disciplines, also the nature of the disability/impairment (congenital versus acquired) was significant. In the multivariate analysis, the nature of disability/impairment was statistically significant, with those with acquired disability reporting lower well-being. Those taking part in wheelchair basketball, para-athletics, and para-swimming competitions had a higher likelihood of reporting well-being, whereas those engaged in wheelchair rugby exhibited lower well-being when compared with controls. An accumulating body of scholarly research carried out on able-bodied athletes and the general population has shown the beneficial impacts of sport and exercise, in terms of improvement in well-being, which, in turn, results in further sports participation and engagement, generating a virtuous circle, with subsequent further enhancements in SWB – the so-called “multiplier effect” theory (Downward et al., 2018). However, the impact on hedonic SWB is less studied. For instance, Wilson et al. (2022) measured the correlation between sports participation and hedonic well-being in adolescents aged 11–17 years, in New Zealand, finding a positive association. However, hedonic SWB was evaluated using a single item rather than a full-length psychometric tool. Hedonic SWB and sports participation have been shown to be mediated by a range of parameters, including age, sex/gender, socio-economic income, relationship status, prior fitness levels, intensity (light versus moderate and high intensity), type (aerobic versus anaerobic and mixed exercise), and duration of physical activity, and sports setting (individual versus team sports) (Ruseski et al., 2014; Wicker and Frick, 2015; Buecker et al., 2021; Habe et al., 2021; Li et al., 2022). Overall, physical activity was found to be related to positive affect, but unrelated to negative affect, enhancing well-being, with effects consistently shown across all age groups and a variety of settings (Buecker et al., 2021). Specifically, regarding competitive sports, some studies (Wann et al., 2015; Fessi et al., 2016; Saw et al., 2016; Watson et al., 2017; Abbott et al., 2018; Fessi and Moalla, 2018; Watson and Brickson, 2018; Watson and Brickson, 2019; Graupensperger et al., 2020; Li et al., 2020; Nobari et al., 2021) identified some associations between well-being and sports-related parameters, like training load, training-induced stress (Saw et al., 2016; Watson and Brickson, 2018; Watson and Brickson, 2019; Nobari et al., 2021), match period (pre-season versus in-season period) (Fessi et al., 2016), match location, match result, and the quality of the opposition during a match (Abbott et al., 2018), and match-induced perceived exertion and fatigue (Fessi and Moalla, 2018), as well as athlete’s identity (Li et al., 2022), social connections, and social identification with college sports teams (Wann et al., 2015; Graupensperger et al., 2020). If the effects of sports engagement on well-being in the able-bodied athlete population is well-known, less has been researched in the disabled athlete community. However, this topic has been attracting increasing scholarly interest, with research documenting that practicing para-sports and participating in events such as the Paralympic Games can contribute to the well-being of disabled individuals. The precise mechanisms are to be elucidated yet, but it can be speculated that a series of direct and indirect, emotional, societal, and motivational factors characterizing the sports arena can, on the one hand, help face challenging circumstances, promote self-acceptance, enhance self-esteem (Vita et al., 2016; Pack et al., 2017), and even reach ambitious goals (Puce et al., 2023). On the other hand, these studies suffer from a few shortcomings, including the use of non-validated, subjective tools, rarely complemented by objective tools (Puce et al., 2023). Positive effects of physical activity and exercise have been shown for a variety of para-sports, like para-swimming (Vita et al., 2016; Pack et al., 2017), and wheelchair sports (Kokaridas et al., 2009; Fiorilli et al., 2013), as well as other paralympic disciplines (Mira et al., 2022). This beneficial impact is, at least partially, counteracted by the competitive nature of the sports environment (Macdougall et al., 2016), characterized by strenuous training schedules (Puce et al., 2018) and organizational-logistical challenges (Hernández-Segura et al., 2022). Besides the positive effects of practicing para-sports, our study found a statistically significant impact of the nature of the disability/impairment (congenital versus acquired). Based on several more recent studies and some earlier studies, whether people were born with or acquired the disability later in life appears to be an important variable when considering psychological well-being. For example, Campbell (1995) compared the psychological well-being associated with congenital versus acquired disability in 93 para-athletes in different sports (track and field, swimming, table tennis, and weightlifting). The authors found that athletes with acquired disabilities reported greater psychological well-being. Additionally, those with a congenital disability had higher anxiety and lower self-control and self-esteem. These results would seem to confirm Wright (1983) findings that people with congenital disabilities in early childhood do not acquire the knowledge and experience necessary to be “independent adults.” Such deficiencies would make these people psychologically fragile and unable to overcome stressful situations. Another possible hypothesis supporting the findings of Campbell (1995) is referred to as the “growth through adversity” theory (Joseph and Linley, 2005; Plews-Ogan et al., 2019). According to this theory, people can experience positive behavioral changes after going through highly stressful adverse events. Thus, adversity can provide opportunities for the development of important character traits such as better relationships with others, identification of new possibilities for one’s life, greater personal strength, spiritual change, and a greater appreciation of life. However, there is also evidence regarding the negative aspects of acquired disabilities. For example, people with recently acquired impairments are prone to lower self-efficacy and show low motivation for competitive sports (Greguol et al., 2015; Veldhuijzen van Zanten et al., 2015; Dehghansai et al., 2020). Thus, although post-traumatic growth is often associated with positive psychological outcomes, it is important to consider that this may occur in parallel with the experience of negative traumatic symptoms yet not fully metabolized (Day, 2013). Providing an explanation and interpretation of our results (acquired disabilities reported lower well-being values) by comparing them with the results of previous studies becomes difficult and cumbersome. The main reason is that we have no data that can attest to how long ago the disability was acquired. Additionally, everyone is unique, which will result in different rehabilitation and athletic pathways regardless of the nature of the disability (Molik et al., 2010; Pinder et al., 2023). Finally, our study documented a sport-specific impact on well-being, with individuals taking part in wheelchair basketball, para-athletics, and para-swimming competitions having a higher likelihood of reporting well-being, different from those engaged in wheelchair rugby, who, in turn, exhibited lower well-being compared with controls. In the Paralympic world, there are sports for athletes with a narrow range of impairments. For example, wheelchair rugby is aimed at people with three or four limb disabilities and was introduced for players with more severe mobility impairments who were unable to participate in wheelchair basketball. Furthermore, Boccia is a sport suitable mainly for people with severe cerebral palsy and spinal cord injuries who would hardly find space in other sports. Also, sports such as blind football, judo, and goalball are sports suitable only for visually impaired athletes. These para-sports appear to induce less well-being than sports such as para-swimming and para-athletics which include a broad spectrum of impairments, including physical, vision, and intellectual disabilities. A possible hypothesis regarding this difference could be given by the fact that the degree and type of functional limitation would become the main criterion for choosing one specific sport over another (Molik et al., 2010). Therefore, the emotions associated with sports, which are the key to a long and rewarding career regardless of competitive results, would be lacking. While we speculate that these factors have an influence and can explain our findings, all these parameters should be formally investigated in future high-quality studies.

The significance and the added value of the present study lie in its ability to provide relevant stakeholders with valuable access to real-life data concerning the well-being of para-athletes. By examining and analyzing this data, researchers and practitioners can gain valuable insights into the specific challenges and factors affecting the quality of life of para-athletes. This knowledge, in turn, serves as a foundation for designing targeted interventions that integrate multiple disciplines, such as sports science, psychology, and rehabilitation. Tailoring these interventions to the unique needs and characteristics of different para-sports disciplines is crucial. Each sport may present distinct physical, psychological, and social demands for athletes, requiring specialized approaches to enhance their well-being and performance.

By considering the specific requirements of each para-sports discipline, multi-disciplinary interventions can be crafted to address the challenges faced by athletes, aiming to optimize their overall quality of life. These interventions may include elements such as physical training programs, psychological support, nutritional guidance, assistive technologies, and social inclusion initiatives. The ultimate goal is to empower para-athletes, helping them overcome barriers, maximize their potential, and lead fulfilling lives both within and beyond the realm of sports. In summary, the practical implications of the present study lie in the utilization of real-life data to inform the design of tailored, multi-disciplinary interventions for para-athletes. By considering the unique needs and characteristics of specific para-sports disciplines, these interventions aim to enhance the well-being and overall quality of life for individuals involved, fostering inclusivity, empowerment, and success in both sports and everyday life.

### 4.1. Limitations and strengths

Limitations of the present study include its cross-sectional study design. Moreover, several para-sports disciplines are not represented and some of those represented may be under-represented. Furthermore, the samples recruited are not representative of the various countries, and it has not been possible to gather data from every European country.

On the other hand, some strengths that should be recognized are the use of validated tools, the large sample size, the cross-country nature of the investigation, the high retention rate of the study, an *a priori* protocol study that was developed and published in a peer-reviewed article (Puce et al., 2017), and a thorough, extensive statistical analysis of the collected data.

## 5. Conclusions and future directions

This study has made a valuable contribution to enhancing our understanding of the well-being of young para-athletes. It highlights that engaging in competitive sports can have a positive impact on their hedonic well-being, which encompasses aspects related to happiness, pleasure, and positive emotions. The findings suggest that participation in sports can be a significant source of enjoyment and fulfillment for youth with disabilities. However, it is important to note that future studies should consider adopting a longitudinal design rather than a cross-sectional approach. While the present study provides valuable insights, a longitudinal design would allow for a more comprehensive examination of the well-being of young para-athletes over an extended period of time. This would enable researchers to investigate how participation in competitive sports impacts various dimensions of well-being, including not only hedonic well-being but also eudaimonic well-being, which pertains to personal growth, self-realization, and a sense of purpose. A longitudinal study would enable researchers to follow para-athletes’ well-being trajectories, capturing changes and fluctuations over time. It could help identify critical periods or developmental milestones when interventions or support systems may be most beneficial. Additionally, a longitudinal approach would allow for the exploration of potential causal relationships between sports participation and well-being, shedding light on the mechanisms through which sports contribute to enhanced well-being among young para-athletes. Such studies would enable researchers and practitioners to develop more targeted interventions and support systems that promote long-term well-being and holistic development among young para-athletes.

## Data availability statement

The original contributions presented in the study are included in the article/supplementary material, further inquiries can be directed to the corresponding authors.

## Ethics statement

The studies involving human participants were reviewed and approved by University of Genoa. Written informed consent to participate in this study was provided by the participants’ legal guardian/next of kin.

## Author contributions

LP, CB, NB, and PR conceived and drafted the manuscript. LP and NB collected and analyzed data. LP, CB, MC, DS, LG, CT, LM, KT, MS, NB, and PR revised the manuscript. All authors contributed to the article and approved the submitted version.

## Conflict of interest

The authors declare that the research was conducted in the absence of any commercial or financial relationships that could be construed as a potential conflict of interest.

## Publisher’s note

All claims expressed in this article are solely those of the authors and do not necessarily represent those of their affiliated organizations, or those of the publisher, the editors and the reviewers. Any product that may be evaluated in this article, or claim that may be made by its manufacturer, is not guaranteed or endorsed by the publisher.

## References

[ref1] Abbott W. Brownlee T. E. Harper L. D. Naughton R. J. Clifford T. (2018). The independent effects of match location, match result and the quality of opposition on subjective wellbeing in under 23 soccer players: a case study. Res. Sports Med. 26, 262–275. doi: 10.1080/15438627.2018.1447476, PMID: 29502448

[ref2] Ascione A. (2018). Sports program to promote the wellbeing of people with disabilities. Acta Medi. Mediterr. 34, 1261–1263. doi: 10.19193/0393-6384_2018_5_194

[ref3] Bahmani B. Mehraban S. Carnero Contentti E. Azkhosh M. Khanjani M. Azimian M. (2022). Relationship of coping strategies with mood symptoms, disease related characteristics and demographic variables in patients with multiple sclerosis: a systematic review study. Mult. Scler. Relat. Disord. 67:104163. doi: 10.1016/j.msard.2022.104163, PMID: 36126537

[ref4] Buecker S. Simacek T. Ingwersen B. Terwiel S. Simonsmeier B. A. (2021). Physical activity and subjective well-being in healthy individuals: a meta-analytic review. Health Psychol. Rev. 15, 574–592. doi: 10.1080/17437199.2020.1760728, PMID: 32452716

[ref5] Campbell E. (1995). Psychological well-being of participants in wheelchair sports: comparison of individuals with congenital and acquired disabilities. Percept. Mot. Skills 81, 563–568. doi: 10.1177/003151259508100241, PMID: 8570358

[ref6] Cooke P. J. Melchert T. P. Connor K. (2016). Measuring well-being: a review of instruments. Couns. Psychol. 44, 730–757. doi: 10.1177/0011000016633507

[ref7] Dantas K. B. A. Dantas E. H. M. Emygdio R. F. Vieira I. B. Reis F. P. (2022). Quality of life of people with motor disabilities, involved or not in parasports. Res. Sq. doi: 10.21203/rs.3.rs-1335577/v1

[ref8] Day M. C. (2013). The role of initial physical activity experiences in promoting posttraumatic growth in paralympic athletes with an acquired disability. Disabil. Rehabil. 35, 2064–2072. doi: 10.3109/09638288.2013.805822, PMID: 23802138

[ref9] Dehghansai N. Lemez S. Wattie N. Pinder R. A. Baker J. (2020). Understanding the development of elite parasport athletes using a constraint-led approach: considerations for coaches and practitioners. Front. Psychol. 11:502981. doi: 10.3389/fpsyg.2020.502981, PMID: 33101110 PMC7554586

[ref10] Diener E. (1984). Subjective well-being. Psychol. Bull. 95, 542–575. doi: 10.1037/0033-2909.95.3.542 6399758

[ref11] Downward P. Hallmann K. Rasciute S. (2018). Exploring the interrelationship between sport, health and social outcomes in the UK: implications for health policy. Eur. J. Pub. Health 28, 99–104. doi: 10.1093/eurpub/ckx063, PMID: 28510694

[ref12] Dupuy H. J. (1984). “The psychological general well-being (PGWB) index. In assessment of quality of life in clinical trials of cardiovascular therapies” in The psychological general well-being (PGWB) index. In assessment of quality of life in clinical trials of cardiovascular therapies. eds. Wenger N. K. Mattson M. E. Furburg C. D. Elinson J. (New York: Le Jacq Publishing), 170–183.

[ref13] Fessi M. S. Moalla W. (2018). Postmatch perceived exertion, feeling, and wellness in professional soccer players. Int. J. Sports Physiol. Perform. 13, 631–637. doi: 10.1123/ijspp.2017-0725, PMID: 29345537

[ref14] Fessi M. S. Nouira S. Dellal A. Owen A. Elloumi M. Moalla W. (2016). Changes of the psychophysical state and feeling of wellness of professional soccer players during pre-season and in-season periods. Res. Sports Med. 24, 375–386. doi: 10.1080/15438627.2016.1222278, PMID: 27574867

[ref15] Filbay S. Pandya T. Thomas B. McKay C. Adams J. Arden N. (2019). Quality of life and life satisfaction in former athletes: a systematic review and meta-analysis. Sports Med. 49, 1723–1738. doi: 10.1007/s40279-019-01163-0, PMID: 31429036 PMC6789047

[ref16] Fiorilli G. Iuliano E. Aquino G. Battaglia C. Giombini A. Calcagno G.. (2013). Mental health and social participation skills of wheelchair basketball players: a controlled study. Res. Dev. Disabil. 34, 3679–3685. doi: 10.1016/j.ridd.2013.08.023, PMID: 24012595

[ref17] Giles S. Fletcher D. Arnold R. Ashfield A. Harrison J. (2020). Measuring well-being in sport performers: where are we now and how do we progress? Sports Med. 50, 1255–1270. doi: 10.1007/s40279-020-01274-z, PMID: 32103451 PMC7305091

[ref18] Graupensperger S. Panza M. J. Budziszewski R. Evans M. B. (2020). Growing into “us”: trajectories of social identification with college sport teams predict subjective well-being. Appl. Psychol. Health Well-Being 12, 787–807. doi: 10.1111/aphw.12207, PMID: 32618406 PMC9750734

[ref19] Greguol M. Gobbi E. Carraro A. (2015). Physical activity practice among children and adolescents with visual impairment – influence of parental support and perceived barriers. Disabil. Rehabil. 37, 327–330. doi: 10.3109/09638288.2014.918194, PMID: 24828394

[ref20] Habe K. Biasutti M. Kajtna T. (2021). Wellbeing and flow in sports and music students during the COVID-19 pandemic. Think. Skills Creat. 39:100798. doi: 10.1016/j.tsc.2021.100798, PMID: 33589864 PMC7874927

[ref21] Hernández-Segura N. Marcos-Delgado A. Pinto-Carral A. Fernández-Villa T. Molina A. J. (2022). Health-related quality of life (HRQOL) instruments and mobility: a systematic review. Int. J. Environ. Res. Public Health 19:16493. doi: 10.3390/ijerph192416493, PMID: 36554369 PMC9779526

[ref22] Iezzoni L. I. (2009). Public health goals for persons with disabilities: looking ahead to 2020. Disabil. Health J. 2, 111–115. doi: 10.1016/j.dhjo.2009.03.002, PMID: 21122749

[ref23] Jefferies P. Gallagher P. Dunne S. (2012). The Paralympic athlete: a systematic review of the psychosocial literature. Prosthet. Orthot. Int. 36, 278–289. doi: 10.1177/0309364612450184 22918904

[ref24] Jones G. Hanton S. Swain A. (1994). Intensity and interpretation of anxiety symptoms in elite and non-elite sports performers. Personal. Individ. Differ. 17, 657–663. doi: 10.1016/0191-8869(94)90138-4

[ref25] Joseph S. Linley P. A. (2005). Positive adjustment to threatening events: an organismic valuing theory of growth through adversity. Rev. Gen. Psychol. 9, 262–280. doi: 10.1037/1089-2680.9.3.262

[ref26] Kim E. S. Chen Y. Nakamura J. S. Ryff C. D. VanderWeele T. J. (2022). Sense of purpose in life and subsequent physical, behavioral, and psychosocial health: an outcome-wide approach. Am. J. Health Promot. 36, 137–147. doi: 10.1177/08901171211038545, PMID: 34405718 PMC8669210

[ref27] Kiuppis F. (2018). Inclusion in sport: disability and participation. Sport Soc. 21, 4–21. doi: 10.1080/17430437.2016.1225882

[ref28] Kokaridas D. Perkos S. Harbalis T. Koltsidas E. (2009). Sport orientation and athletic identity of Greek wheelchair basketball players. Percept. Mot. Skills 109, 887–898. doi: 10.2466/pms.109.3.887-898, PMID: 20178288

[ref29] Krahn G. L. Walker D. K. Correa-De-Araujo R. (2015). Persons with disabilities as an unrecognized health disparity population. Am. J. Public Health 105, S198–S206. doi: 10.2105/AJPH.2014.302182, PMID: 25689212 PMC4355692

[ref30] Li B. Ding C. Fan F. Shi H. Guo L. Yang F. (2020). Associations between psychological profiles and performance success among professional taekwondo athletes in China: a multidimensional scaling profile analysis. Front. Psychol. 11:822. doi: 10.3389/fpsyg.2020.00822, PMID: 32499737 PMC7242764

[ref31] Li W. Zhang L. Li C. Zhu N. Zhao J. Kong F. (2022). Pursuing pleasure or meaning: a cross-lagged analysis of happiness motives and well-being in adolescents. J. Happiness Stud. 23, 3981–3999. doi: 10.1007/s10902-022-00576-5

[ref32] Lundqvist C. (2011). Well-being in competitive sports—the feel-good factor? A review of conceptual considerations of well-being. Int. Rev. Sport Exerc. Psychol. 4, 109–127. doi: 10.1080/1750984X.2011.584067

[ref33] Lundqvist C. Sandin F. (2014). Well-being in elite sport: dimensions of hedonic and Eudaimonic well-being among elite orienteers. Sport Psychol. 28, 245–254. doi: 10.1123/tsp.2013-0024

[ref34] Macdougall H. O’Halloran P. Sherry E. Shields N. (2016). Needs and strengths of Australian Para-athletes: identifying their subjective psychological, social, and physical health and well-being. Sport Psychol. 30, 1–12. doi: 10.1123/tsp.2015-0006

[ref35] Macdougall H. O’Halloran P. Shields N. Sherry E. (2015). Comparing the well-being of para and Olympic sport athletes: a systematic review. Adapt. Phys. Act. Q. 32, 256–276. doi: 10.1123/APAQ.2014-0168, PMID: 26113553

[ref36] Mack D. E. Wilson P. M. Gunnell K. E. Gilchrist J. D. Kowalski K. C. Crocker P. R. E. (2012). Health-enhancing physical activity: associations with markers of well-being. Appl. Psychol. Health Well Being 4, 127–150. doi: 10.1111/j.1758-0854.2012.01065.x, PMID: 26286974

[ref37] Maugeri G. Castrogiovanni P. Battaglia G. Pippi R. D’Agata V. Palma A.. (2020). The impact of physical activity on psychological health during Covid-19 pandemic in Italy. Heliyon 6:e04315. doi: 10.1016/j.heliyon.2020.e04315, PMID: 32613133 PMC7311901

[ref38] McLoughlin E. Fletcher D. Slavich G. M. Arnold R. Moore L. J. (2021). Cumulative lifetime stress exposure, depression, anxiety, and well-being in elite athletes: a mixed-method study. Psychol. Sport Exerc. 52:101823. doi: 10.1016/j.psychsport.2020.101823, PMID: 33281503 PMC7710337

[ref39] Mira T. Monteiro D. Costa A. M. Morouço P. Matos R. Antunes R. (2022). Tokyo 2020: a sociodemographic and psychosocial characterization of the Portuguese paralympic team. Healthcare 10:1185. doi: 10.3390/healthcare10071185, PMID: 35885712 PMC9316738

[ref40] Molik B. Zubala T. Słyk K. Bigas G. Gryglewicz A. Kucharczyk B. (2010). Motivation of the disabled to participate in chosen paralympics events (wheelchair basketball, wheelchair rugby, and boccia). Physiotherapy 18, 42–51. doi: 10.2478/v10109-010-0044-5

[ref41] Nobari H. Alves A. R. Haghighi H. Clemente F. M. Carlos-Vivas J. Pérez-Gómez J.. (2021). Association between training load and well-being measures in young soccer players during a season. Int. J. Environ. Res. Public Health 18:4451. doi: 10.3390/ijerph18094451, PMID: 33922250 PMC8122726

[ref42] Pack S. Kelly S. Arvinen-Barrow M. (2017). “I think I became a swimmer rather than just someone with a disability swimming up and down:” paralympic athletes perceptions of self and identity development. Disabil. Rehabil. 39, 2063–2070. doi: 10.1080/09638288.2016.1217074, PMID: 27673551

[ref43] Paleari F. G. Pivetti M. Galati D. Fincham F. D. (2021). Hedonic and eudaimonic well-being during the COVID-19 lockdown in Italy: the role of stigma and appraisals. Br. J. Health Psychol. 26, 657–678. doi: 10.1111/bjhp.12508, PMID: 33460503 PMC8013861

[ref44] Pinder R. Dehghansai N. Baker J. (2023). “Talent development opportunities and challenges in paralympic sport: an introduction” in Talent development in paralympic sport: researcher and practitioner perspectives. eds. Dehghansai N. Pinder R. A. Baker J. (New York and London: Routledge)

[ref45] Plews-Ogan M. Ardelt M. Owens J. (2019). Growth through adversity: exploring associations between internal strengths, posttraumatic growth, and wisdom. J. Value Inq. 53, 371–391. doi: 10.1007/s10790-018-9659-4

[ref46] Puce L. Marinelli L. Girtler N. G. Pallecchi I. Mori L. Simonini M.. (2019). Self-perceived psychophysical well-being of young competitive swimmers with physical or intellectual impairment. Percept. Mot. Skills 126, 862–885. doi: 10.1177/0031512519865849 31362582

[ref47] Puce L. Marinelli L. Mori L. Pallecchi I. Trompetto C. (2017). Protocol for the study of self-perceived psychological and emotional well-being of young Paralympic athletes. Health Qual. Life Outcomes 15:219. doi: 10.1186/s12955-017-0798-2, PMID: 29132378 PMC5683577

[ref48] Puce L. Marinelli L. Pierantozzi E. Mori L. Pallecchi I. Bonifazi M.. (2018). Training methods and analysis of races of a top level paralympic swimming athlete. J. Exerc. Rehabil. 14, 612–620. doi: 10.12965/jer.1836254.127, PMID: 30276182 PMC6165978

[ref49] Puce L. Okwen P. M. Yuh M. N. Akah Ndum Okwen G. Pambe Miong R. H. Kong J. D.. (2023). Well-being and quality of life in people with disabilities practicing sports, athletes with disabilities, and para-athletes: insights from a critical review of the literature. Front. Psychol. 14:1071656. doi: 10.3389/fpsyg.2023.1071656, PMID: 36844305 PMC9945540

[ref50] Rice S. M. Purcell R. De Silva S. Mawren D. McGorry P. D. Parker A. G. (2016). The mental health of elite athletes: a narrative systematic review. Sports Med. 46, 1333–1353. doi: 10.1007/s40279-016-0492-2, PMID: 26896951 PMC4996886

[ref51] Ruseski J. E. Humphreys B. R. Hallman K. Wicker P. Breuer C. (2014). Sport participation and subjective well-being: instrumental variable results from German survey data. J. Phys. Act. Health 11, 396–403. doi: 10.1123/jpah.2012-0001, PMID: 23363531

[ref52] Ryan R. M. Deci E. L. (2001). On happiness and human potentials: a review of research on hedonic and Eudaimonic well-being. Annu. Rev. Psychol. 52, 141–166. doi: 10.1146/annurev.psych.52.1.141, PMID: 11148302

[ref53] Saw A. E. Main L. C. Gastin P. B. (2016). Monitoring the athlete training response: subjective self-reported measures trump commonly used objective measures: a systematic review. Br. J. Sports Med. 50, 281–291. doi: 10.1136/bjsports-2015-094758, PMID: 26423706 PMC4789708

[ref54] Tilly L. (2008). Enabling people with learning disabilities to manage their own health and well-being. Med. Confl. Surviv. 24, S108–S113. doi: 10.1080/13623690801957471, PMID: 18771202

[ref55] Tough H. Siegrist J. Fekete C. (2017). Erratum to: social relationships, mental health and wellbeing in physical disability: a systematic review. BMC Public Health 17:580. doi: 10.1186/s12889-017-4448-8, PMID: 28622768 PMC5473103

[ref56] Trigueros R. Pérez-Jiménez J. M. García-Mas A. Aguilar-Parra J. M. Fernandez-Batanero J. M. Luque de la Rosa A.. (2021). Adaptation and validation of the Eudaimonic well-being questionnaire to the Spanish sport context. Int. J. Environ. Res. Public Health 18:3609. doi: 10.3390/ijerph18073609, PMID: 33807158 PMC8036994

[ref57] Vandenbroucke J. P. Poole C. Schlesselman J. J. Egger M. (2007). Strengthening the reporting of observational studies in epidemiology (STROBE): explanation and elaboration. PLoS Med. 18, 805–835. doi: 10.1097/EDE.0b013e3181577511 18049195

[ref58] Veldhuijzen van Zanten J. J. C. S. Rouse P. C. Hale E. D. Ntoumanis N. Metsios G. S. Duda J. L.. (2015). Perceived barriers, facilitators and benefits for regular physical activity and exercise in patients with rheumatoid arthritis: a review of the literature. Sports Med. 45, 1401–1412. doi: 10.1007/s40279-015-0363-2, PMID: 26219268 PMC4579262

[ref59] Vita G. La Foresta S. Russo M. Vita G. L. Messina S. Lunetta C.. (2016). Sport activity in Charcot–Marie–tooth disease: a case study of a paralympic swimmer. Neuromuscul. Disord. 26, 614–618. doi: 10.1016/j.nmd.2016.06.002, PMID: 27460291 PMC5026044

[ref60] Wann D. L. Waddill P. J. Brasher M. Ladd S. (2015). Examining sport team identification, social connections, and social well-being among high school students. J. Amat. Sport 1, 27–50. doi: 10.17161/jas.v0i0.4931

[ref61] Watson A. Brickson S. (2018). Impaired sleep mediates the negative effects of training load on subjective well-being in female youth athletes. Sports Health 10, 244–249. doi: 10.1177/1941738118757422, PMID: 29420135 PMC5958455

[ref62] Watson A. Brickson S. (2019). Relationships between sport specialization, sleep, and subjective well-being in female adolescent athletes. Clin. J. Sport Med. 29, 384–390. doi: 10.1097/JSM.0000000000000631, PMID: 31460952

[ref63] Watson A. Brickson S. Brooks A. Dunn W. (2017). Subjective well-being and training load predict in-season injury and illness risk in female youth soccer players. Br. J. Sports Med. 51, 194–199. doi: 10.1136/bjsports-2016-096584, PMID: 27919919

[ref64] Wicker P. Frick B. (2015). The relationship between intensity and duration of physical activity and subjective well-being. Eur. J. Pub. Health 25, 868–872. doi: 10.1093/eurpub/ckv131 26142405

[ref65] Wilson O. W. A. Whatman C. Walters S. Keung S. Enari D. Chiet A.. (2022). “Balance is better”: the wellbeing benefits of participating in a breadth of sports across a variety of settings during adolescence. Int. J. Environ. Res. Public Health 19:8597. doi: 10.3390/ijerph19148597, PMID: 35886452 PMC9322196

[ref66] Wright B. A. (1983). Physical disabilities: a psychological approach. New York: Harper & Row.

